# Epigenetic regulation of adaptive responses of forest tree species to the environment

**DOI:** 10.1002/ece3.461

**Published:** 2013-01-17

**Authors:** Katharina Bräutigam, Kelly J Vining, Clément Lafon-Placette, Carl G Fossdal, Marie Mirouze, José Gutiérrez Marcos, Silvia Fluch, Mario Fernández Fraga, M Ángeles Guevara, Dolores Abarca, Øystein Johnsen, Stéphane Maury, Steven H Strauss, Malcolm M Campbell, Antje Rohde, Carmen Díaz-Sala, María-Teresa Cervera

**Affiliations:** 1 Centre for the Analysis of Genome Evolution and Function, Department of Cell & Systems Biology, University of TorontoToronto, ON, M5S 3B2, Canada; 2 Department of Forest Ecosystems and Society, Oregon State UniversityCorvallis, OR, 97331-5752, USA; 3 UFR-Faculté des Sciences, UPRES EA 1207 ‘Laboratoire de Biologie des Ligneux et des Grandes Cultures’ (LBLGC), INRA, USC1328 ‘Arbres et Réponses aux Contraintes Hydrique et Environnementales’ (ARCHE), University of OrléansRue de Chartres, BP 6759, F-45067, Orléans, France; 4 Department of Biology and Environment, Norwegian Forest and Landscape InstitutePO Box 115, N-1431, Aas, Norway; 5 Epigenetic Regulations and Seed Development, Institut de Recherche pour le Développement, UMR232 ERL5300 CNRS-IRD911 Av. Agropolis, 34394, Montpellier, France; 6 School of Life Sciences, University of WarwickWellesbourne, Warkwick, CV35 9EF, United Kingdom; 7 Platform for Integrated Clone Management (PICME), Health & Environment Department, AIT Austrian Institute of Technology GmbHKonrad-Lorenz-Straße 24, 3430, Tulln, Austria; 8 Cancer Epigenetics Laboratory, Institute of Oncology of Asturias (IUOPA–HUCA), University of OviedoSpain; 9 Dpt. of Forest Ecology and Genetics, Forest Genomics and Ecophysiology group, Forest Research Centre (CIFOR). INIACrta. La Coruña km 7,5, 28040, Madrid, Spain; 10 Mixed Unit of Forest Genomics and Ecophysiology, INIA/UPMMadrid, Spain; 11 Department of Life Sciences, University of AlcaláCtra. Madrid-Barcelona Km. 33,600, 28871, Alcalá de Henares, Madrid, Spain; 12 Department of Plant and Environmental Sciences, Norwegian University of Life SciencesPO Box 5003, N-1432, Ås, Norway; 13 Department of Biological Sciences, University of Toronto Scarborough, University of Toronto1265 Military Trail, Toronto, ON, M1C 1A4, Canada; 14 Department Plant Growth & Development, Institute of Agriculture and Fisheries ResearchCaritasstraat 21, 9090, Melle, Belgium

**Keywords:** Adaptive response, environmental stress, epigenetic memory of stressful conditions, epigenetics, forest trees, phenotypic plasticity

## Abstract

Epigenetic variation is likely to contribute to the phenotypic plasticity and adaptative capacity of plant species, and may be especially important for long-lived organisms with complex life cycles, including forest trees. Diverse environmental stresses and hybridization/polyploidization events can create reversible heritable epigenetic marks that can be transmitted to subsequent generations as a form of molecular “memory”. Epigenetic changes might also contribute to the ability of plants to colonize or persist in variable environments. In this review, we provide an overview of recent data on epigenetic mechanisms involved in developmental processes and responses to environmental cues in plant, with a focus on forest tree species. We consider the possible role of forest tree epigenetics as a new source of adaptive traits in plant breeding, biotechnology, and ecosystem conservation under rapid climate change.

## Introduction

Epigenetics refers to the study of meiotically or mitotically heritable changes in gene function that do not result from changes in DNA sequence (Bonasio et al. 2010). At the molecular level, epigenetic phenomena are mediated by reversible marks such as DNA methylation and histone modifications, and by small RNAs that can alter regulatory states of genes or genomic regions. DNA methylation in plants occurs at cytosines in all sequence contexts (CG, CHG, and CHH where H = A, T, or C), and well-studied post-translational modifications of histone proteins at specific amino acid residues include methylation (Krauss 2008), acetylation, phosphorylation (Demidov et al. 2009) and ubiquitination (Kouzarides 2007). Genome-wide epigenetic patterns, referred to as “epigenomes”, are not static; rather, they can undergo precise changes. Epigenome modifications are involved in biological processes including genetic imprinting, transposon silencing, regulation of gene expression, and heterochromatin organization.

The influence of environmental factors on epigenetic marks, and on the resultant changes in gene expression and phenotype, has recently attracted considerable attention (Boyko and Kovalchuk 2008; Chinnusamy and Zhu 2009; Feil and Fraga 2011; Groszmann et al. 2011a; Mirouze and Paszkowski 2011). Knowledge of the regulatory mechanisms involved in adaptive epigenetic responses may help to guide management of genetic resources and plant breeding, especially in long-lived forest tree species where changes in allele frequency are expected to occur very slowly. This review provides a brief overview of recent data on epigenetic mechanisms involved in developmental processes and responses to environmental cues in forest species, as well as the implications of forest tree epigenetics to adaptation as a possible new source of beneficial traits for plant breeding and conservation in ecosystems responding to climate change.

## Factors driving epigenetic regulation in plants

### Epigenetic regulation in plant development

Epigenetic regulation plays important roles in multiple aspects of plant development. Two distinct roles of this regulation can be distinguished, depending on whether they concern developmentally regulated genes or transposable elements (TEs). In developmentally regulated genes, epigenetic marks allow switches in gene expression in response to developmental transitions and/or environmental cues. After sexual reproduction, uniparental expression of parental alleles, imprinting, is associated with discrete differentially methylated regions (DMRs) that act in a genome context-independent manner (Gutierrez-Marcos et al. 2006; Jullien et al. 2006; Haun et al. 2007).

A well-characterized example of epigenetic control during post-embryonic development is vernalization, the phenomenon of cold temperature-induced competence to flower (Chouard 1960; Schmitz and Amasino 2007). In *Arabidopsis thaliana*, regulation of *FLOWERING LOCUS C (FLC)* gene expression during vernalization illustrates how environmental cues are perceived and translated into epigenetic marks that affect plant development (Bastow et al. 2004; Heo and Sung 2011; Kim and Sung 2012).

The epigenetic marks of many loci involved in plant development are normally erased or reset at each generation following meiosis, thus preventing the establishment of new “epialleles” (alleles whose expression is conditioned by their epigenetic status). On the other hand, stable, that is, heritable, epialleles can occur naturally and might confer specific phenotypes. Examples in plants include floral symmetry in *Linaria* that is influenced by DNA methylation levels at the *CYCLOIDEA* locus (Cubas et al. 1999) and absence of ripening in tomato, that is associated with hypermethylation at the *Colorless non-ripening* locus (Manning et al. 2006). Stable epialleles are potential targets for selection in evolutionary processes, or in applied plant breeding. More examples will contribute to a better understanding of their origin, their stability, and the role they might play in selection.

In contrast to the transient nature of many developmental epigenetic marks, those affecting TEs are more stable (Slotkin and Martienssen 2007; Bourc'his and Voinnet 2010; Lisch and Slotkin 2011) and the mobility of TEs is observed when these marks are alleviated in mutants affected in the epigenetic machinery (Mirouze et al. 2009; Tsukahara et al. 2009; Ito et al. 2011). However, during development, transcription of activated TEs in hypomethylated gamete companion cells is thought to produce small RNAs that migrate into the germ cell and direct the silencing machinery to TEs. Hence, at each new generation, the “immune system” against transposons is perpetuated, but also readjusted to prevent potential genome invasion of new mobile elements (Lisch and Slotkin 2011). Given the abundance of TEs in tree genomes, they should be considered as potential sources of epigenetic variation potentially affecting regulation of nearby genes.

The importance of developmentally related epigenetic modification has been underscored recently by its potential involvement in hybrid vigor. Hybrid vigor, also known as heterosis, describes the superior performance of hybrid progeny over their parents in traits like biomass and seed production or stress resistance. Various models explaining heterotic effects at single loci have been proposed, including dominance, overdominance, and pseudo-overdominance, while interactions between genes (epistasis) have been considered as well (Birchler et al. 2010). The molecular mechanisms causing non-additive gene expression in hybrids have been the focus of studies in rice and *A. thaliana*, and epigenetic regulation has recently been associated with heterosis (Ha et al. 2009; He et al. 2010; Groszmann et al. 2011a,b). In hybrids, a number of short interfering RNAs (siRNAs) were found to accumulate to non-additive levels, which in turn can lead to changes in DNA methylation and gene expression, thus contributing to hybrid vigor (Groszmann et al. 2011a,b). Given the preponderance of hybrids in many plant taxa, including prominent tree genera like *Populus*, the putative involvement of epigenetics in heterosis is of great interest.

### Epigenetic regulation in plant environmental responses

Various environmental signals and stresses can induce persistent changes in epigenetic modifications, thereby creating a flexible “memory” system for short or prolonged periods of time (Kvaalen and Johnsen 2008; Chinnusamy and Zhu 2009; Jablonka and Raz 2009; Whittle et al. 2009). Environmental conditions have an impact on a number of different epigenetic marks and mechanisms, including DNA methylation and histone modifications, or on frequencies of homologous recombination and genomic rearrangements (Bond and Finnegan 2007; Chinnusamy and Zhu 2009; Feil and Fraga 2011; Hauser et al. 2011; Mirouze and Paszkowski 2011). For example, changes in genome-wide DNA methylation patterns in response to biotic and abiotic stress treatments (pathogen, herbivore, high salt, low nutrients) occur in asexually reproduced dandelions (*Taraxacum officinale*). Notably, altered DNA methylation patterns were transmitted to the non-stressed progeny in this species and the potential role of stress-induced epigenetic inheritance in evolution has been discussed (Verhoeven et al. 2010). The involvement of a histone variant (H2A.Z) was found to mediate short-term adaptation to temperature change in *A. thaliana* (Kumar and Wigge 2010), and cold stress-induced hypomethylation and transposition of a TE (Tam-3) has been observed in *Antirrhinum* (Hashida et al. 2006).

Epigenetic recombinant inbred lines (epiRILs) have emphasized the relationship between response to environmental conditions and epigenetic phenomena. In *A. thaliana,* epiRILs have nearly identical genomes, but display diverse mosaic epigenomes with variant DNA methylation patterns (Johannes et al. 2009; Reinders et al. 2009). The range of pathogen sensitivity/resistance within one isogenic epiRIL population exhibited 60% of the range of pathogen responses observed in natural, genetically varying *A*. *thaliana* accessions (Reinders et al. 2009). In the context of environmental challenges, such epigenetic modifications may be thought of as relatively “plastic” yet heritable marks that allow for rapid responses and adaptations and, at the same time, might avoid excessive genetic diversification (Boyko and Kovalchuk 2008; Lira-Medeiros et al. 2010).

## Epigenetic control in forest tree species

### Relationship between epigenetic and phenotypic plasticity

Forest trees are long-lived organisms with complex life cycles, which must contend with a variable environment over their long lifetimes (Rohde and Junttila 2008). The long generation times impose limits on natural selection under rapidly changing climate conditions (Rehfeldt et al. 1999, 2002). Consequently, trees must be highly adaptable, displaying a wide range of phenotypes as a function of their environments, known as phenotypic plasticity (Nicotra et al. 2010). Phenotypic plasticity is likely to be of great importance for both individual trees and forest populations over near- and long-term timescales. Despite this, knowledge of the extent and underlying mechanisms of phenotypic plasticity in response to a variety of stress responses and developmental traits in trees is rudimentary (Rohde 2009; Lira-Medeiros et al. 2010; Neale and Kremer 2011).

In addition to the genetic component, epigenetic variation has been suggested to contribute to the phenotypic plasticity and adaptive potential of individuals and populations (Bossdorf et al. 2008; Jablonka and Raz 2009; Herrera and Bazaga 2010; Lira-Medeiros et al. 2010; Richards et al. 2012). Insight into epigenetic variation, and its relationship to phenotypic plasticity, will contribute to the understanding of adaptive plant responses, and might help to evaluate the risk of long-lived species to both short-term and long-term fluctuations in the environment. Moreover, understanding the interplay between epigenetics and adaptation should enhance the understanding of evolutionary trajectories, as natural selection also directly targets the proportion of phenotypic variation that is shaped by epigenetic phenomena (Bossdorf et al. 2008; Herrera and Bazaga 2010).

### Epigenetic and phenotypic variation in natural populations, ecotypes, and species

Despite the substantial impact that epigenetics might have in determining environmental compatibility, relatively few studies have investigated the extent of natural epigenetic variation and its relationship to phenotypic variation and adaptation potential (Cervera et al. 2002; Bossdorf et al. 2008; Jablonka and Raz 2009; Marfil et al. 2009; Herrera and Bazaga 2010; Lira-Medeiros et al. 2010; Paun et al. 2010). Among the different epigenetic mechanisms that are potentially involved in transgenerational inheritance and natural epigenetic variation, DNA methylation represents the most-studied modification (Akimoto et al. 2007; Jablonka and Raz 2009; Herrera and Bazaga 2010; Lira-Medeiros et al. 2010; Paun et al. 2010; Verhoeven et al. 2010). Two of the few published studies in higher plants that considered the interplay between genetic, epigenetic, as well as phenotypic variation and environmental factors, focused on a perennial violet species (*Viola cazorlensis*) and orchids of the *Dactylorhiza majalis* complex (Herrera 1990; Herrera and Bazaga 2010; Paun et al. 2010). The studies detected coordinated genetic–epigenetic adaptive differentiation, indicating the involvement of epigenetic processes in adaptation and evolution by influencing primary phenotypic diversity.

In tree species, natural variation in epigenetic marks and the relation to phenotypic traits is still an under-explored area. Insight into the role of epigenetics in determining tree phenotype should identify key elements in the control of growth traits and contribute to the understanding of evolutionary capacity of tree species (Grattapaglia et al. 2009; Thumma et al. 2009; Lira-Medeiros et al. 2010). In keeping with this, evidence for the correlation between tree form and epigenetics is emerging. Trees of the white mangrove (*Laguncularia racemosa*) can occur naturally in contrasting habitats and can exhibit striking differences in morphological traits (Lira-Medeiros et al. 2010). Tree-like appearance was documented in a riverside habitat with abundant fresh water and nutrient supply, whereas in a nearby salt marsh habitat, mangrove plants were characterized by abnormal growth and shrub-like morphology. Notably, despite morphological dissimilarities, analysis of DNA nucleotide sequences and methylation patterns detected greater epigenetic than genetic variation within and between populations in contrasting environments, which indicates that epigenetic variation in natural populations plays an important role in long-term adaptation to different environments (Lira-Medeiros et al. 2010).

**Figure 1:**
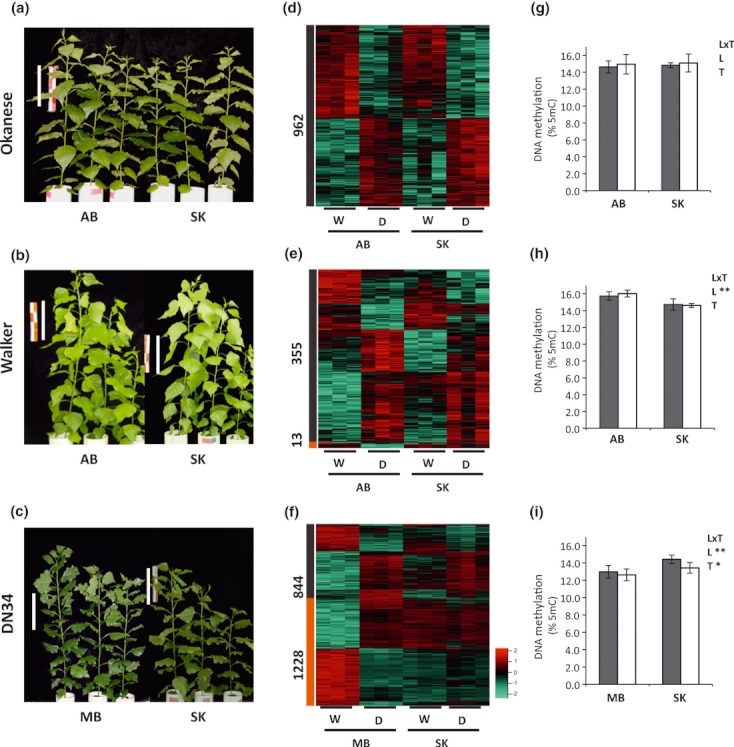
Clone history shapes drought responses in poplar hybrids. Transcriptome-level responses to water withholding are influenced by geographic origin for two of the three genotypes, and are paralleled by differences in total (genome-wide) DNA methylation. Ramets of hybrid poplar genotypes (a) Okanese, (b) Walker, and (c) DN34 with distinct histories were obtained from two different locations for each of the genotypes. The response to water deficit was assessed under common, controlled environmental conditions. Okanese (a, d, g); Walker (b, e, h); DN34 (c, f, i). Tree appearance (a–c). Transcriptome-level responses (d–f). Heat maps represent relative abundance of drought responsive transcripts at pre-dawn for Okanese (d), Walker (e), and Okanese (f) obtained from two locations each. The numbers indicated to the side of the heat map correspond to transcripts with significant treatment main effect only (gray) and with significant treatment: location interactions (orange bar) (BH adjusted, *P* < 0.05). W, well-watered samples; D, water-deficient samples. Global DNA methylation levels as percentage of 5 mC under well-watered (shaded bars) and water-limited conditions (white bars) for the genotypes Okanese (g), Walker (h), and DN34 (l). L, location effect; T, treatment effect; and LxT, location: treatment interaction term (*P < 0.05, **P < 0.001, *n* = 6, SD bars). Locations are abbreviated as follows: AB, Alberta; SK, Saskatchewan; MB, Manitoba. Figure is adapted from Raj et al. 2011.

The lasting impact of previous environmental history on a tree's capacity to respond to a current environmental stimulus has recently been explored in *Populus* (Raj et al. 2011). Poplar trees are frequently propagated vegetatively through stem cuttings of branches containing dormant buds, generating genetically identical individuals or ramets of the same genotype. Clonally propagated poplar trees can be planted in different geographic locations, thus giving rise to populations of genetically identical ramets that are characterized by their own local environment and history. To study the lasting effect of clone history on current plant performance, cuttings of the same genotype were obtained from different geographic locations and grown under common environmental conditions, after which the transcriptome response to an important environmental stress, drought, was studied. Notably, differences in transcript abundance patterns in response to drought that were based on differences in geographic origin of clonally propagated trees were detected in two of the three investigated genotypes. These transcriptome-level patterns were paralleled by differences in genome-wide DNA methylation. Genotypes with the longest time since establishment and last common propagation showed the most pronounced location-specific patterns in transcriptome response and DNA methylation indicating a possible epigenomic basis for clone history-dependent transcriptome divergence (Fig. 1). These findings underline the importance of epigenetic mechanisms related to the adaptation of long-lived species like poplar trees to the local environment (Raj et al. 2011).

**Figure 2:**
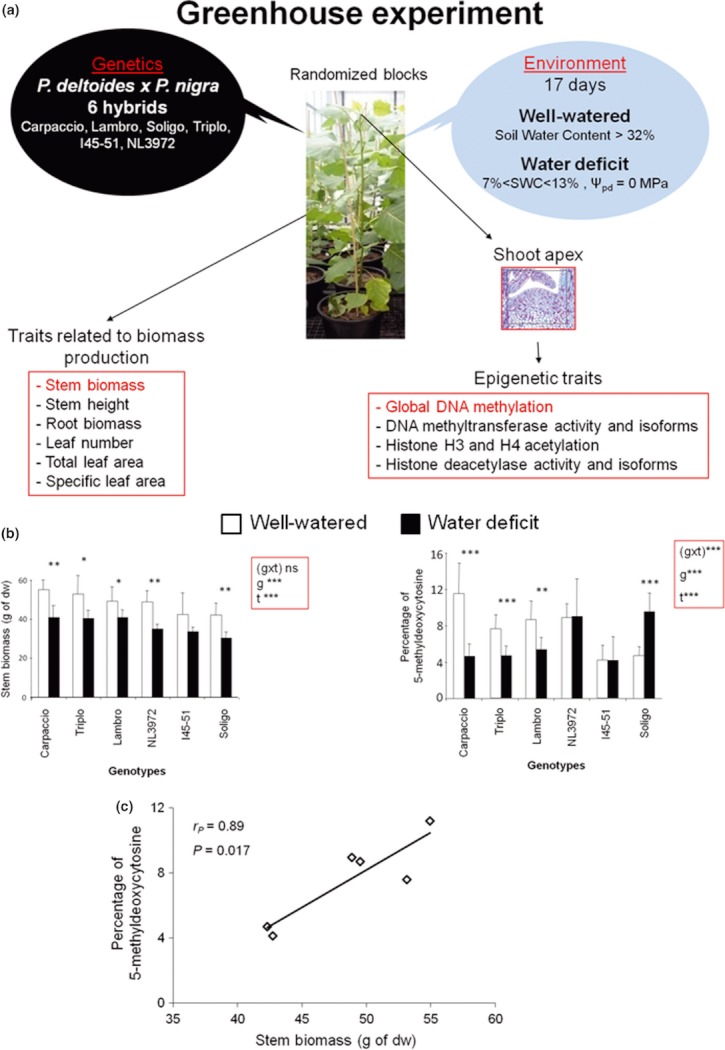
Relation between epigenetic marks and the genotypic variability of phenotypic plasticity under limited water availability or not in six poplar hybrids. (a). Experimental design; (b). Stem biomass and DNA methylation levels in the shoot apex (center of morphogenesis). For each graph, g indicates the genotype effect, t the treatment effect, and (gxt) genotype by treatment effect. Means are accompanied by their standard errors SE (*n* = 6). Significant differences between well-watered and water-deficit conditions are indicated by asterisk: *P ≤ 0.05, **P ≤ 0.01, and ***P ≤ 0.001; c. Linear correlation (Pearson, *r*) between stem biomass and DNA methylation levels. Adapted from Gourcilleau et al. 2010.

The direct response of six hybrid poplar genotypes to water deficit revealed a relationship between epigenetic marks and the genotypic variability of phenotypic plasticity (Gourcilleau et al. 2010). Genotypic variation for both DNA methylation and traits related to biomass productivity was observed in hybrids (*Populus deltoids* × *P. nigra*), and a positive correlation was established among these variables in well-watered conditions (Fig. 2). While poplar genotypes showed reduced growth in water-deficit conditions, a significant genotype effect was observed for DNA methylation variations. This suggests that DNA methylation could participate in the fine-tuning of gene expression in poplar during water stress (Plomion et al. 2006; Bogeat-Triboulot et al. 2007; Bonhomme et al. 2009; Wilkins et al. 2009; Gourcilleau et al. 2010; Hamanishi and Campbell 2011).

The potential link between natural epigenetic variation and phenotypic variability observed in trees is further supported by studies in ecotypes and individual populations of specific herbaceous plant species (Cervera et al. 2002; Marfil et al. 2009). Highly conserved DNA methylation patterns were detected within an *A. thaliana* ecotype (L*er*) while clear DNA methylation differences existed between ecotypes that did not correlate with nucleotide sequence variation, but with their flowering time (Cervera et al. 2002). Furthermore, variation in the floral phenotype of individuals from a single natural population of a wild hybrid potato (*Solanum ruiz-lealii*) was found to correlate with distinct DNA methylation patterns, but not with DNA sequence variation (Marfil et al. 2009).

Most studies assessing epigenetic variation in natural populations, ecotypes, or species focused on the extent of epigenetic variability and paid less attention to the functional consequences. Indication for a functional link between a specific epigenetic mark at a specific position in the genome and variation in a quantitative trait was discovered by analyzing polymorphisms in an association population and a full-sib family of eucalypt (*Eucalyptus nitens;* Thumma et al. 2009). Making use of the low linkage disequilibrium in populations of forest trees, variation in cellulose content was linked to polymorphisms within a gene potentially involved in cellulose synthesis and deposition (functional polymorphisms). The COBRA-like gene *En*COBL4A was strongly associated with a QTL region for cellulose content and fine mapping revealed a significant association with a SNP in exon 5. Notably, allelic expression imbalance was linked to allele-specific cytosine methylation upstream of this SNP in a full-sibling family. A heritable epigenetic polymorphism is thus likely to influence phenotypic variation in cellulose content; however, further functional analyses are required (Thumma et al. 2009). The findings suggest that epigenetic variations might contribute to quantitative trait variation (Thumma et al. 2009), and it has been suggested that this phenomenon might be common (Johannes et al. 2008; Reinders et al. 2009; Thumma et al. 2009; Long et al. 2011).

To date, some prominent, shared observations have emerged from the few studies of natural epigenetic variation and phenotypic plasticity. These studies established that (1) epigenetic variation occurs in natural populations, ecotypes, and species; (2) this variation can correlate with naturally occurring phenotypic variation; and (3) there is a potential role for epigenetic variation in adaptation and potentially in evolution. Despite the commonalities that have emerged from these studies, many questions remain unresolved. For example, is epigenetic variation in natural populations a wide-spread phenomenon? Moreover, the key molecular mechanisms involved and how they are regulated remain to be determined. Finally, it remains unclear to what extent epialleles arise and how stable they are when considered in an evolutionary context (Bossdorf et al. 2008; Herrera and Bazaga 2010; Lira-Medeiros et al. 2010; Paun et al. 2010). Answers to such questions might also contribute to a better understanding of the adaptive capability of long-lived forest trees that might help to assess their susceptibility to rapidly changing environments (Grattapaglia et al. 2009).

### Epigenetic plasticity in growth and development

During their relatively long lifespans, trees must make developmental adjustments while retaining flexibility to match and synchronize growth and development with prevailing environmental conditions. Epigenetic mechanisms are proposed to contribute to such flexible adjustments by generating transmittable and reversible marks that constitute temporary “memory” systems (Boyko and Kovalchuk 2008; Kvaalen and Johnsen 2008; Yakovlev et al. 2010; Jaskiewicz et al. 2011). Tissue-, organ-, and species-specific differences in DNA methylation levels are well known (Fraga et al. 2002a,b; Valledor et al. 2007, 2010; Monteuuis et al. 2009; Santamaria et al. 2009; Rodriguez Lopez et al. 2010; Vining et al. 2012; Lafon-Placette et al. 2013. Changes in epigenetic marks were found to accompany morphological and physiological changes in trees in a wide variety of processes, including aging, phase change, organ maturation, and bud set or burst (Fraga et al. 2002a,b; Santamaria et al. 2009; Valledor et al. 2010).

Bud dormancy is a vital adaptation to seasonal changes, and release and induction of bud dormancy are complex processes that largely determine length of the growth season, and thereby affect annual tree productivity. Regulation of bud burst integrates endogenous and exogenous signals such as hormone levels, day length, light quality, and temperature (Santamaria et al. 2009) and involves substantial changes in gene expression and epigenetic modifications (Ruttink et al. 2007; Rohde 2009; Santamaria et al. 2009). In apical buds of a chestnut (*Castanea sativa*), a decrease in global DNA methylation level and concomitant increase in acetylation of histone 4 were observed during bud burst when conditions were favorable for active growth. The opposite pattern (i.e., DNA hypermethylation and lower histone acetylation levels), indicative of more repressive chromatin states, was detected during bud set when environmental conditions were less favorable for growth (Santamaria et al. 2009). The observed coordinated changes in DNA methylation and histone modifications are predicted to alter the control of gene expression to shape the processes of bud burst and bud set (Santamaria et al. 2009).

Aging and maturation are characterized by altered patterns of cell differentiation and organ formation processes, and the potential role of DNA methylation in maturation has been studied in some tree species (Fraga et al. 2002a,b; Valledor et al. 2007; Monteuuis et al. 2009). For example, studies in radiata pine (*Pinus radiata*) support the involvement of DNA methylation in this process. Changes in global DNA methylation levels of up to 25% during maturation have been reported in this species (Fraga et al. 2002a,b). In juvenile plants without flowering capability, young needle tissue was characterized by a markedly lower extent of DNA methylation than corresponding tissues in adult trees with reproductive ability. Regarding histone modifications, decreased levels of euchromatin-associated marks, such as histone 4 acetylation and specific histone methylation (trimethylation of histone 3 on lysine 4 or H3K4me3) have been measured in mature needles when compared with juvenile ones (Valledor et al. 2010). Moreover, the observed increase in DNA methylation levels from juvenile to mature plants in meristematic tissue could be directly linked to phase change. Conversely, an increase in the degree of tree reinvigoration by serial grafting, measured by the recovery of morphogenetic competence, was accompanied by a decrease in global level of DNA methylation in meristematic tissue, thus pointing toward plasticity of DNA methylation marks during aging and maturation. The degree of DNA methylation, as well as additional biochemical characteristics, was proposed to serve as suitable markers for aging and reinvigoration in pine (Fraga et al. 2002a,b). However, differences between species and experimental systems might exist. In another conifer, *Larix laricina*, age-related changes in foliar traits were observed, whereas differences in DNA methylation levels between juvenile and mature scions could not be detected in DNA from whole needles (Greenwood et al. 1989).

In angiosperms, heteroblastic tree species like *Acacia mangium* with distinct leaf morphologies of juvenile and mature stages provide excellent systems to study aging. Small but significant differences between microshoots with juvenile (pinnate) and mature (phyllode) morphology were observed in this acacia species when analyzing global DNA methylation levels in physiologically active apical buds of *in vitro* grown plant material. Here, the degree of DNA methylation was higher in juvenile than in mature tissue, and might be influenced by *in vitro* culture conditions (see Epigenetic and phenotypic plasticity in artificial systems) in addition to maturation-related processes (Baurens et al. 2004; Monteuuis et al. 2009). Taken together, the aforementioned studies establish a clear relationship between DNA methylation levels and maturation for some tissue types and species in woody plants (Fraga et al. 2002a,b; Baurens et al. 2004; Valledor et al. 2007; Monteuuis et al. 2009). Observed differences might be attributable to differences in taxonomy, tissue type (meristematic vs. differentiated), or experimental system (*in vitro*, field conditions) and might also reflect underlying mechanistic differences in the relationship between aging and epigenetic marks (Fraga et al. 2002b; Monteuuis et al. 2009). Furthermore, the data indicate that DNA methylation patterns are not static and can exhibit remarkable dynamics and plasticity during development and seasonal changes (Fraga et al. 2002b; Valledor et al. 2007; Monteuuis et al. 2009).

**Figure 3:**
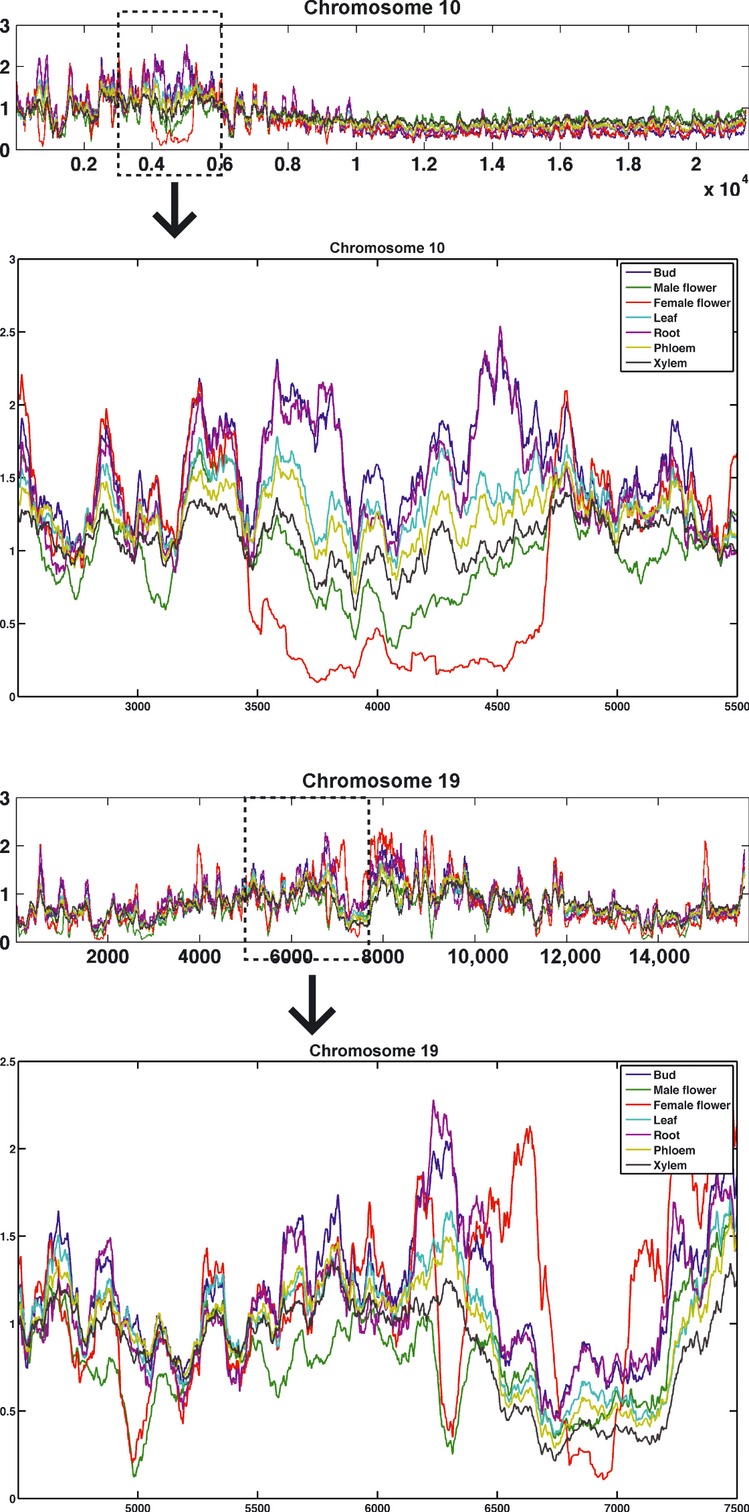
DNA methylation profiles vary widely among chromosomes and among tissues at selected loci in *Populus trichocarpa*. Relative DNA methylation was determined using methylated DNA immunoprecipitation followed by Illumina sequencing (MeDIP-seq). The ratio of MeDIP-seq read counts in immunoprecipitated (IP) samples versus non-IP control is plotted in 1-kb windows for chromosomes 10 and 19, and areas of tissue-differential methylation are expanded below each chromosome. Figures from Vining et al. (2012).

Evidence for the remarkable dynamics and plasticity of epigenetic modification in tree species is growing. Genome-level comparative analysis of cytosine methylation among differentiated poplar tissues revealed highly heterogeneous DNA methylation profiles among chromosomes, and a number of cases of tissue-specific methylation (Fig. 3; Vining et al. 2012), many of them associated with gene bodies or promoters. Although a broadly similar chromosome methylation and gene expression profile was observed in poplar when compared to *A. thaliana* and other plant species, significant differences were also detected. For example, only in poplar was gene body (i.e., the entire gene from the transcription start site to the end of the transcript) methylation associated with greater repression of gene expression than was promoter methylation. In addition, Vining et al. (2012) observed a distinctive pattern of transposon and gene body methylation for male catkins compared with other tissues, including female catkins. Recently, analysis of the methylome of open chromatin in poplar meristematic cells found that 74% of poplar gene models had gene body methylation, and its intensity, as well as cytosine context, varied depending on gene size, redundancy in the genome (presence of paralogs), and extent of tissue-specific gene expression (Lafon-Placette et al. 2013).

Plasticity in tree epigenetic modification has also been observed in conifer species, specifically as it relates to phenology. Phenology responses of seedlings that were produced in warm or cold years vary within the same stands (Kohmann and Johnsen 1994). In Norway spruce, a temperature-dependent epigenetic “memory” from the time of embryo development, which thereafter influences the timing of bud phenology and gene expression, has been discovered (Skrøppa and Johnsen 2000; Johnsen et al. 2005; Yakovlev et al. 2010). Colder-than-normal conditions during embryogenesis and seed development advance the timing, whereas temperatures above normal delay the onset of these adaptive processes, and the altered performance is long lasting in the progeny. This phenomenon was initially discovered when ecotypes from northern Norway were transferred to a southern seed orchard where they produced progenies with a phenology similar to that of southern ecotypes (Johnsen et al. 1996; Skrøppa and Johnsen 2000). Notably, differences in day length and temperature applied during pollen formation did not affect the progeny performance. Differences in the female flowering environment did affect progeny performance. The temperature during zygotic embryogenesis and seed maturation shifted the developmental program of the seeds, resulting in significant phenotypic changes, with the effect lasting as long as over 20 years (Skrøppa and Johnsen 2000; Skrøppa et al. 2010; Yakovlev et al. 2010). The traits that are affected include the timing of dehardening and bud burst in the spring; leader shoot growth cessation in the summer and bud set and cold acclimation in the autumn. All processes are thus advanced or delayed as influenced by the temperature during reproduction in progeny with identical genetic background. Similar effects have been observed in progeny from white spruce (*Picea glauca* × *Picea engelmannii*) crosses, Scots pine, *Larix* spp., and longleaf pine (Dormling and Johnsen 1992, 1992; Greenwood and Hutchison 1996; Stoehr et al. 1998; Webber et al. 2005), but there is lack of information regarding this phenomenon in angiosperm trees (Rohde and Junttila 2008). In birch (*Betula pendula*), a small-scale study within a population revealed a close genetic relationship between trees that had established in a year of similar temperature (Kelly et al. 2003).

The importance of plastic epigenetic modification on phenology in conifer species extends beyond the individual to encompass the ecosystem. Epigenetic effects taking place during zygote development may create phenotypic diversity at the local community level, if temperature varies considerably among successive generations. This is particularly important as phenology traits are strongly genetically differentiated.

The molecular mechanism behind this striking epigenetic “memory” phenomenon is not yet clear, but transcriptional changes have been implicated (Johnsen et al. 2005; Yakovlev et al. 2010, 2011). In progeny that differ epigenetically, transcriptional analysis revealed that seedlings from full-sib families produced at different embryogenesis temperatures under long- and short-day conditions differed. Suppressive subtracted cDNA libraries revealed considerable differences in their transcriptomes. MicroRNA pathway genes *DICER-LIKE1* (*PaDCL1*), *DICER-LIKE 2* (*PaDCL2*), and *SUPPRESSOR OF GENE SILENCING 3-LIKE* (*PaSGS3*), as well as transposon-related genes, had altered transcript abundance in epigenetically different progeny with phenotypic differences in bud burst and bud set (Yakovlev et al. 2011). Norway spruce contains a set of conserved miRNAs as well as a large proportion of novel non-conserved miRNAs involved in temperature-dependent epigenetic “memory”. Most of the miRNAs were targeted to previously unknown genes, or genes with no known function. The expression of seven conserved and nine novel miRNAs showed significant differences in transcript levels in progenies showing distinct epigenetic difference in bud set, but not in the progeny from a non-responding family without differences in bud set, making them excellent candidate miRNAs. The altered transcript abundance of specific miRNAs suggests their putative participation in epigenetic regulation (Yakovlev et al. 2010). This epigenetic phenomenon is not only generated in controlled Norway spruce crosses, but such epitypes can also be produced by somatic embryogenesis (Kvaalen and Johnsen 2008). Genetically identical plants generated at different temperatures by zygotic embryogenesis expressed a difference in timing of terminal bud formation that was equivalent to a 4–6° latitudinal ecotypic difference.

The “memory” effects acting on phenological traits lasted for more than 20 years after germination and affected long-term growth under field conditions (Skrøppa et al. 2007). Notably, there was absence of any genetic segregation distortion in the progeny, strongly supporting that this “memory”, affecting the climatic adaptation in this species, is indeed an epigenetic phenomenon (Besnard et al. 2008). Thus, distinct epitypes can be produced from the same genotype in Norway spruce, a process not well documented in other tree species so far. In view of rapid climate change, strategies to increase diversity for selection might be of prime importance for survival of species within their current geographic distribution, and therefore this epigenetic “memory” mechanism is likely of evolutionary significance and has obvious practical implications.

### Epigenetic and phenotypic plasticity in artificial systems

While epigenetic phenomena are clearly important for trees in a natural context, they also could be of great consequence during specific tree production processes integrated into the wood products chain. Long generation times and the out-crossing habit of a number of forest trees can make it difficult to rapidly propagate material and maintain valuable genotypes under natural conditions. Tissue culture can provide alternative means to keep desirable genotypes by vegetative propagation and to quickly produce commercial quantities of regenerants; therefore, micropropagation is widely used in forestry. It has been observed, however, that tissue culture can introduce variation in regenerated plants. This somaclonal variation can result in subtle to drastic phenotypic variation and has been found to be attributable to genetic or epigenetic variations (e.g., reviewed in Kaeppler et al. 2000; Miguel and Marum 2011). Somaclonal variation (heritable across mitotic and meiotic cell divisions) has been considered both beneficial and disadvantageous (Jaligot et al. 2000; Kaeppler et al. 2000; Schellenbaum et al. 2008), and a number of studies have focused on elucidating underlying mechanisms (Kaeppler et al. 2000; Rival et al. 2008; Schellenbaum et al. 2008; Rodriguez Lopez et al. 2010).

A well-studied example for somaclonal variants and their relation to epigenetic marks in a tree species is the *mantled* phenotype in somatic-embryo-derived oil palm (*Elaeis guineensis*). This phenotypic variant, found in about five percent of regenerants, is characterized by abnormal inflorescence development and has been associated with global DNA hypomethylation, but not to changes in genomic structure or nucleotide sequence (Jaligot et al. 2000; Rival et al. 2008). The exact mechanisms involved in generating somaclonal variants like the mantled phenotype remain largely unresolved. Ongoing studies of this phenomenon might help to better understand mechanisms of epigenetic responses to tissue-culture-induced stresses (Kaeppler et al. 2000; Rival et al. 2008).

It has also been observed that the ability to generate mature somatic embryos from cultured tissue can decrease as a culture ages and that somaclonal variation can increase with culture age (Phillips et al. 1994; Valledor et al. 2007; Krizova et al. 2009). In addition to other mechanisms, changes in DNA methylation were considered to contribute to the reduction in embryonic potential or organogenic potential in tissue culture and grafting procedures (Fraga et al. 2002b; Valledor et al. 2007). A detailed analysis of genetic and epigenetic variation in relation to callus age reports interesting plasticity in cocoa plants (*Theobroma cacao*) regenerated by somatic embryogenesis. Genetic variation was investigated using single sequence repeat (SSR) markers, and epigenetic variability was assessed by methylation-sensitive amplified polymorphism (MSAP), a method to detect genome-wide but anonymous DNA methylation patterns. Contrary to predictions, after an initial increase, a decrease in both genetic and epigenetic divergence between leaves of regenerants and the ortet plant was observed after the culture had reached an age of about 10 weeks (Rodriguez Lopez et al. 2010). One possible interpretation of the findings suggests a link between stable DNA methylation patterns and repression of *de novo* mutations during somatic embryogenesis (Rodriguez Lopez et al. 2010).

For many plant species, different physiological and developmental stages of diverse tissue explant types have been associated with distinct epigenetic characteristics, in particular DNA methylation (Fraga et al. 2002a,b; Monteuuis et al. 2009; Santamaria et al. 2009; Rodriguez Lopez et al. 2010; Valledor et al. 2010). For example, some DNA methylation patterns and levels, characteristics of the source tissue used to start an *in vitro* culture, were retained in regenerants in acacia and cocoa (Monteuuis et al. 2009; Rodriguez Lopez et al. 2010). This highlights the plasticity of DNA methylation marks under tissue culture conditions. Transitions from juvenile to adult phase are frequently accompanied by reduction or loss of morphogenetic ability in woody species (see Epigenetic plasticity in growth and development). Concomitant with maturation of pine needles, changes in epigenetic marks were measured when compared with immature needles. This finding could be in accordance with a less permissive and reprogrammable chromatin state and could account in part for the reduced organogenic capacity of explants from mature needles.

Generation of somaclonal genetic and epigenetic variants as well as plasticity in DNA methylation is widely documented outcomes of plant regeneration in tissue culture (Kaeppler et al. 2000; Marfil et al. 2009). Studying underlying mechanisms might be of relevance for basic research and applications in plant propagation such as the understanding of differentiation and dedifferentiation processes or the selection of appropriate *in vitro* culture conditions (Kaeppler et al. 2000; Marfil et al. 2009; Rodriguez Lopez et al. 2010).

## Strategic and technical approaches to study epigenetic processes

### Selection of appropriate systems

Different methods have been used in model plants to analyze epigenetic variation independently of genetic variation. These have included treatment with demethylating agents, analysis of natural epimutations, and study of DNA methylation-deficient mutants. Epigenetic recombinant inbred lines (epiRILs) have been developed in *A. thaliana* (Johannes et al. 2009; Reinders et al. 2009) using isogenic lines (wild types and mutant lines) differing only in the level and distribution of DNA methylation (see Epigenetic regulation in plant environmental responses). These lines represent a powerful tool to identify specific epigenomic regions that are associated with the observed phenotypic variation through epiQTL mapping approaches that are based on methylation-sensitive markers. The epiQTL mapping approach requires the establishment of multiple plant generations, and may be difficult to apply to tree species that require a significant amount of time to reach sexual maturation.

To discern genetic and epigenetic effects, clonally propagated plants or systems that are characterized by reduced genetic variation, such as stone pine (*Pinus pinea*), represent ideal study subjects. To separate heritable from non-heritable epigenetic variation (resulting from developmental plasticity in response to different environments), it is necessary to study, when available, clonally propagated genotypes, the progeny of different natural populations or maternal families in a common environment, and to use the resemblance of epigenetic patterns among relatives as an indication of epigenetic inheritance (Bossdorf et al. 2008).

### Technical approaches

A wide variety of techniques have been developed to study epigenetic patterns and modifications. Histone modifications can be analyzed by chromatin immunoprecipation (ChIP) using antibodies that recognize specific histone modifications, followed by either microarray hybridization (ChIP on chip) or by next generation sequencing (ChIP-Seq; Ku et al. 2011). DNA methylation at the genome level, the DNA methylome, can be investigated by methylated DNA immunoprecipitation (meDIP) or by bisulfite treatment of the DNA followed by hybridization to a microarray, or by next generation sequencing (BS-Seq; Ku et al. 2011; Krueger et al. 2012; Cokus et al. 2008). Additionally, direct detection of methylated residues using DNA synthesis technologies based on variable polymerase kinetics depending on the chemical modification of the template nucleotide (e.g., 5-methylcytosine *vs*. cytosine) represents a novel method to directly detect DNA methylation (Flusberg et al. 2010).

Next generation sequencing technologies enable mapping of epigenetic modifications at single-base resolution. The nature and large amount of data generated by such technologies will demand new approaches in data analysis techniques. Inference of the methylation status of bisulfite-treated DNA by BS-Seq can be challenging as the data obtained do not exactly match the reference sequence. Consequently, both DNA strands must be considered separately, and methylation at a specific site can be a percentage rather than a total presence or absence. Nevertheless, a number of tools has been developed to facilitate these analyses and are now available for application to tree epigenomes (Chen et al. 2010; Lim et al. 2010; Krueger et al. 2012).

## Conclusions

Many questions remain about the mechanisms and roles of epigenetic processes in enabling rapid adaptation of plants to their environment, especially in forest trees. Recently, genome-wide studies of chromatin-bound proteins and epigenetic marks in *Drosophila melanogaster* and in *A. thaliana* have substantially revised our understanding of chromatin (Roudier et al. 2011; Van Steensel 2011). The dogma of an uncompacted, transcriptionally active euchromatin *versus* a compacted, silent heterochromatin is likely to be an oversimplification of the real chromatin architecture. It appears that chromatin might be composed of several types differing in their epigenetic marks as well as in their nuclear localization and chromatin-associated proteins. These types could favor or prevent association with transcription factors, thus defining gene expression patterns. Whether these chromatin types exist in perennial species is not known, and the stability of these chromatin types in long-living organisms is to be established. Similarly, the maintenance of these types during clonal and *in vitro* culture propagation will give important clues about the effect of these biotechnologies on gene expression control.

It has been observed that the induction of alternative epigenetic states not only triggers the formation of new epialleles but also promotes the movement of DNA transposons and retroelements that are very abundant in plant genomes (Mirouze and Paszkowski 2011). However, mechanisms counteracting accumulation of induced epialleles must also be in place, because otherwise we would be “constantly confronted with the inheritance of environmentally induced phenotypic variation” (Richards 2006). Additionally, in large genomes, such as those of conifer [with C estimates of DNA content ranging from 17 to 30 Gbp for pines and spruces of which more than 68% are attributed to repeated DNA (Rake et al. 1980; Ohri and Khoshoo 1986)], cytosine methylation is implicated in genomic compartmentalization, *that is*, non-coding highly repeated sequences get separated from low-copy sequence and transcriptionally active regions. The differential methylation of genic and non-genic regions observed across plant taxa may be involved in decreasing transcriptional “noise” (Rabinowicz et al. 2005). In large genomes, epigenetic mechanisms might be more prominent, as a means to control the repetitive parts of the genome. This might render their entire genomes more amenable to epigenetic regulation.

From an economic and ecological point of view, it is important to integrate information on epigenetic control of environmental and developmental processes in both forest resources management and breeding. In quantitative genetic studies, estimates of genetic variance over the total phenotypic variance are typically used to assess the heritability of a trait. Akin to other genetic characters, variance in epigenetic characters will contribute to genetic variance and/or phenotypic variance, but might go undetected in some studies, or might be confounded with normal Mendelian-based quantitative inheritance (Kalisz and Purugganan 2004). Epigenetic effects may thus inflate the true genetic variation in traits. As a consequence, the genetic clines observed for many phenology traits, even in common garden experiments, may reflect more local adaptation than DNA sequence-based genetic differences among populations.

Recent developments show that both energy efficiency and energy homeostasis, which are integral parts of yield, have an epigenetic component that can be directed and stabilized by artificial selection (i.e., selective breeding; De Block and Van Lijsebettens 2011). These findings open new possibilities for engineering plant metabolism and improving complex traits. For example, in addition to the unintended genetic and epigenetic variation imparted by *in vitro* manipulation, it may be considered and utilized as a means to amplify or release epigenetic variation of value to breeding programs. Transgenic perturbation of epigenetic mechanisms might have similar effects; however, testing such effects using a transgenic approach with forest trees at a scale relevant to application and ecological variation are, at present, constrained by government regulations (Viswanath et al. 2012).

Genome perturbation, including epigenetic components, might be important for increasing the raw material for adaptive evolution under severe stress (Kalisz and Purugganan 2004; Rapp and Wendel 2005). Rapp and Wendel (2005) suggest that a population bottleneck, while reducing genetic diversity, might simultaneously create epigenetic novelty. In contrast to genetic alleles, epialleles might react more quickly to environmental change, be reversible, and persist for only a few generations (Kalisz and Purugganan 2004). If a new epiallele were to cause a mild phenotype through alteration of gene expression, it might experience less strong selection than a loss-of-function sequence mutation (Kalisz and Purugganan 2004) and thus enable rapid, yet fine-tuned, trait modifications. The significance of epialleles in wild populations will depend on their frequency and stability (Rohde and Junttila 2008).

The analysis of the epigenetic processes in an ecological context, known as “ecological epigenetics” is set to transform our understanding of the way in which organisms function on the landscape. Forest trees offer excellent opportunities to examine some of the most compelling questions of ecological epigenetics (Bossdorf et al. 2008), particularly those related to the interplay between epigenetic variation and phenotypic variation in natural populations, and the role of epigenetic variation in evolutionary processes. Ecological epigenetics could readily address such questions by capitalizing on the advantageous features of forest trees, including their long lifespans, their dominance of many ecosystems, their wide geographic distribution, and their life histories, especially reproductive traits like clonal propagation. Analysis of the epigenetics of forest tree species will significantly improve our understanding of the mechanisms underlying natural phenotypic variation, and the responses of organisms to environmental change, and may thereby inform efforts to manage and breed tree species to help them cope with environmental stresses.

## References

[b1] Akimoto K, Katakami H, Kim HJ, Ogawa E, Sano CM, Wada Y (2007). Epigenetic inheritance in rice plants. Ann. Bot.

[b2] Bastow R, Mylne JS, Lister C, Lippman Z, Martienssen RA, Dean C (2004). Vernalization requires epigenetic silencing of FLC by histone methylation. Nature.

[b3] Baurens FC, Nicolleau J, Legavre T, Verdeil JL, Monteuuis O (2004). Genomic DNA methylation of juvenile and mature *Acacia mangium* micropropagated in vitro with reference to leaf morphology as a phase change marker. Tree Physiol.

[b4] Besnard G, Acheré V, Jeandroz S, Johnsen Ø, Faivre Rampant P, Baumann R (2008). Does maternal environmental condition during reproductive development induce genotypic selection in *Piceaabies*. Ann. For. Sci.

[b5] Birchler JA, Yao H, Chudalayandi S, Vaiman D, Vieti RA (2010). Heterosis. Plant Cell.

[b6] Bogeat-Triboulot MB, Brosché M, Renaut J, Jouve L, Fayyaz D, Le Thiec P (2007). Gradual soil water depletion results in reversible changes of gene expression, protein profiles, ecophysiology, and growth performance in *Populus euphratica*, a poplar growing in arid regions. Plant Physiol.

[b7] Bonasio R, Tu S, Reinberg D (2010). Molecular signals of epigenetic states. Science.

[b8] Bond DM, Finnegan EJ (2007). Passing the message on: inheritance of epigenetic traits. Trends Plant Sci.

[b9] Bonhomme L, Monclus R, Vincent D, Carpin S, Lomenech AM, Plomion C (2009). Leaf proteome analysis of eight *Populus* x *euramericana* genotypes: genetic variation in drought response and in water-use efficiency involves photosynthesis-related proteins. Proteomics.

[b10] Bossdorf O, Richards CL, Pigliucci M (2008). Epigenetics for ecologists. Ecol. Lett.

[b11] Bourc'his D, Voinnet O (2010). A Small-RNA perspective on gametogenesis, fertilization, and early zygotic development. Science.

[b12] Boyko A, Kovalchuk I (2008). Epigenetic control of plant stress response. Environ. Mol. Mutagen.

[b13] Cervera MT, Ruiz-Garcia L, Martínez-Zapater JM (2002). Analysis of DNA methylation in *Arabidopsis thaliana* based on methylation-sensitive AFLP markers. Mol. Genet. Genomics.

[b14] Chen PY, Cokus SJ, Pellegrini MBS (2010). Seeker: precise mapping for bisulfite sequencing. BMC Bioinformatics.

[b15] Chinnusamy V, Zhu JK (2009). Epigenetic regulation of stress responses in plants. Curr. Opin. Plant Biol.

[b16] Chouard P (1960). Vernalization and its relations to dormancy. Annu. Rev. Plant Physiol.

[b17] Cokus SJ, Feng S, Zhang X, Chen Z, Merriman B, Haudenschild CD (2008). Shotgun bisulphite sequencing of the Arabidopsis genome reveals DNA methylation patterning. Nature.

[b18] Cubas P, Vincent C, Coen E (1999). An epigenetic mutation responsible for natural variation in floral symmetry. Nature.

[b19] De Block M, Van Lijsebettens M (2011). Energy efficiency and energy homeostasis as genetic and epigenetic components of plant performance and crop productivity. Curr. Opin. Plant Biol.

[b20] Demidov D, Hesse S, Tewes A, Rutten T, Fuchs J, Ashtiyani RK (2009). Aurora1 phosphorylation activity on histone H3 and its cross-talk with other post-translational histone modifications in Arabidopsis. Plant J.

[b21] Dormling I, Johnsen Ø (1992). Effects of the parental environment on full-sib families of *Pinus sylvestris*. Can. J. For. Res.

[b22] Feil R, Fraga MF (2011). Epigenetics and the environment: emerging patterns and implications. Nat. Rev. Genet.

[b23] Flusberg BA, Webster DR, Lee JH, Travers KJ, Olivares EC, Tyson AC (2010). Direct detection of DNA methylation during single-molecule, real-time sequencing. Nat. Methods.

[b24] Fraga MF, Canal MJ, Rodriguez R (2002a). Phase-change related epigenetic and physiological changes in *Pinus radiata* D. Don. Planta.

[b25] Fraga MF, Rodriguez R, Canal MJ (2002b). Genomic DNA methylation-demethylation during aging and reinvigoration of *Pinus radiata*. Tree Physiol.

[b26] Gourcilleau D, Bogeat-Triboulot MB, Lafon-Placette D, Le Thiec C, Delaunay A, El-Soud WA (2010). DNA methylation and histone acetylation: genotypic variations in hybrid poplars, impact of water deficit and relationships with productivity. Ann. For. Sci.

[b27] Grattapaglia D, Plomion C, Kirst M, Sederoff RR (2009). Genomics of growth traits in forest trees. Curr. Opin. Plant Biol.

[b28] Greenwood MS, Hutchison KW, Hom J, Birdsey R, O'Brian K (1996). Genetic after effects of increased temperature in *Larix*. Proceedings of the 1995 Meeting of the Northern Global Change Program.

[b29] Greenwood MS, Hopper CA, Hutchison KW (1989). Maturation in larch: I. Effect of age on shoot growth, foliar characteristics, and DNA methylation. Plant Physiol.

[b30] Groszmann M, Greaves IK, Albert N, Fujimoto R, Helliwell CA, Dennis ES (2011a). Epigenetics in plants-vernalisation and hybrid vigour. Biochim. Biophys. Acta.

[b31] Groszmann M, Greaves IK, Albertyn ZI, Scofield GN, Peacock WJ, Dennis ES (2011b). Changes in 24-nt siRNA levels in Arabidopsis hybrids suggest an epigenetic contribution to hybrid vigor. Proc. Natl Acad. Sci. USA.

[b32] Gutierrez-Marcos JF, Costa LM, Dal Prà M, Scholten S, Kranz E, Perez P (2006). Epigenetic asymmetry of imprinted genes in plant gametes. Nat. Genet.

[b33] Ha M, Lu J, Tian L, Ramachandran V, Kasschau KD, Capman EJ (2009). Small RNAs serve as a genetic buffer against genomic shock in Arabidopsis interspecific hybrids and allopolyploids. Proc. Natl Acad. Sci. USA.

[b34] Hamanishi ET, Campbell MM (2011). Genome-wide responses to drought in forest trees. Forestry.

[b35] Hashida SN, Uchiyama T, Martin C, Kishima Y, Sano Y, Mikami T (2006). The temperature-dependent change in methylation of the Antirrhinum transposon Tam3 is controlled by the activity of its transposase. Plant Cell.

[b36] Haun WJ, Laoueille-Duprat S, O'Connell MJ, Spillane C, Grossniklaus U, Phillips AR (2007). Genomic imprinting, methylation and molecular evolution of maize Enhancer of zeste (Mez) homologs. Plant J.

[b37] Hauser M, Aufsatz W, Jonak C, Luschnig C (2011). Transgenerational epigenetic inheritance in plants. Biochim. Biophys. Acta.

[b38] He G, Zhu X, Elling AA, Chen L, Wang X, Guo L (2010). Global epigenetic and transcriptional trends among two rice subspecies and their reciprocal hybrids. Plant Cell.

[b39] Heo JB, Sung S (2011). Vernalization-mediated epigenetic silencing by a long intronic noncoding RNA. Science.

[b40] Herrera CM (1990). The adaptedness of the floral phenotype in a relict endemic, hawkmoth-pollinated violet. 2. Patterns of variation among disjunct populations. Biol. J. Linn. Soc.

[b41] Herrera CM, Bazaga P (2010). Epigenetic differentiation and relationship to adaptive genetic divergence in discrete populations of the violet *Viola cazorlensis*. New Phytol.

[b42] Ito H, Gaubert H, Bucher E, Mirouze M, Vaillant I, Paszkowski J (2011). An siRNA pathway prevents transgenerational retrotransposition in plants subjected to stress. Nature.

[b43] Jablonka E, Raz G (2009). Transgenerational epigenetic inheritance: prevalence, mechanisms, and implications for the study of heredity and evolution. Q. Rev. Biol.

[b44] Jaligot E, Rival A, Beule T, Dussert S, Verdeil JL (2000). Somaclonal variation in oil palm (*Elaeis guineensis* Jacq.): the DNA methylation hypothesis. Plant Cell Rep.

[b45] Jaskiewicz M, Conrath U, Peterhansel C (2011). Chromatin modification acts as a memory for systemic acquired resistance in the plant stress response. EMBO Rep.

[b46] Johannes F, Colot V, Jansen RC (2008). Epigenome dynamics: a quantitative genetics perspective. Nat. Rev. Genet.

[b47] Johannes F, Porcher E, Teixeira FK, Saliba-Colombani V, Simon M, Agier N (2009). Assessing the impact of transgenerational epigenetic variation on complex traits. PLoS Genet.

[b48] Johnsen Ø, Skrøppa T, Junttila O, Dæhlen OG (1996). Influence of the female flowering environment on autumn frost-hardiness of *Picea abies* progenies. Theor. Appl. Genet.

[b49] Johnsen Ø, Fossdal CG, Nagy N, Molmann J, Dælen OG, Skrøppa T (2005). Climatic adaptation in *Picea abies* progenies is affected by the temperature during zygotic embryogenesis and seed maturation. Plant Cell Environ.

[b50] Jullien PE, Kinoshita T, Ohad N, Berger F (2006). Maintenance of DNA methylation during the Arabidopsis life cycle is essential for parental imprinting. Plant Cell.

[b51] Kaeppler SM, Kaeppler HF, Rhee Y (2000). Epigenetic aspects of somaclonal variation in plants. Plant Mol. Biol.

[b52] Kalisz S, Purugganan MD (2004). Epialleles via DNA methylation: consequences for plant evolution. Trends Ecol. Evol.

[b53] Kelly CK, Chase MW, Fay A, De Bruijn MF, Woodward FI (2003). Temperature-based population segregation in birch. Ecol. Lett.

[b54] Kim DH, Sung S (2012). Environmentally coordinated epigenetic silencing of FLC by protein and long noncoding RNA components. Curr. Opin. Plant Biol.

[b55] Kohmann K, Johnsen Ø (1994). The timing of bud-set in seedlings of *Picea abies* from seed crops of a cool versus a warm summer. Silvae Genetica.

[b56] Kouzarides T (2007). Chromatin modifications and their function. Cell.

[b57] Krauss V (2008). Glimpses of evolution: heterochromatic histone H3K9 methyltransferases left its marks behind. Genetica.

[b58] Krizova K, Fojtova M, Depicker A, Kovarik A (2009). Cell culture-induced gradual and frequent epigenetic reprogramming of invertedly repeated tobacco transgene epialleles. Plant Physiol.

[b59] Krueger F, Kreck B, Franke A, Andrews SR (2012). DNA methylome analysis using short bisulfite sequencing data. Nat. Methods.

[b60] Ku CS, Naidoo N, Wu M, Soong R (2011). Studying the epigenome using next generation sequencing. J. Med. Genet.

[b61] Kumar SV, Wigge PA (2010). H2A.Z-containing nucleosomes mediate the thermosensory response in Arabidopsis. Cell.

[b62] Kvaalen H, Johnsen Ø (2008). Timing of bud set in *Picea abies* is regulated by a memory of temperature during zygotic and somatic embryogenesis. New Phytol.

[b63] Lafon-Placette C, Faivre-Rampant P, Delaunay A, Street N, Brignolas F, Maury S (2013). Methylome of DNase I sensitive chromatin in Populus trichocarpa shoot apical meristematic cells: a simplified approach revealing characteristics of gene-body DNA methylation in open chromatin state. New Phytol.

[b64] Lim SJ, Tan TW, Tong JC (2010). Computational epigenetics: the new scientific paradigm. Bioinformation.

[b65] Lira-Medeiros CF, Parisod C, Fernandes RA, Mata CS, Cardoso MA, Ferreira PC (2010). Epigenetic variation in mangrove plants occurring in contrasting natural environment. PLoS ONE.

[b66] Lisch DR, Slotkin RK (2011). Strategies for silencing and escape: the ancient struggle between transposable elements and their hosts. Int. Rev. Cell Mol. Biol.

[b67] Long Y, Xia W, Li R, Wang J, Shao M, Feng J (2011). Epigenetic QTL mapping in Brassica napus. Genetics.

[b68] Manning K, Tör M, Poole M, Hong Y, Thompson AJ, King GJ (2006). A naturally occurring epigenetic mutation in a gene encoding an SBP-box transcription factor inhibits tomato fruit ripening. Nat. Genet.

[b69] Marfil CF, Camadro EL, Masuelli RW (2009). Phenotypic instability and epigenetic variability in a diploid potato of hybrid origin, *Solanum ruiz-lealii*. BMC Plant Biol.

[b70] Miguel C, Marum L (2011). An epigenetic view of plant cells cultured in vitro: somaclonal variation and beyond. J. Exp. Bot.

[b71] Mirouze M, Paszkowski J (2011). Epigenetic contribution to stress adaptation in plants. Curr. Opin. Plant Biol.

[b72] Mirouze M, Reinders J, Bucher E, Nishimura T, Schneeberger K, Ossowski S (2009). Selective epigenetic control of retrotransposition in *Arabidopsis*. Nature.

[b73] Monteuuis O, Baurens FC, Goh DKS, Doulbeau S, Verdeil JL (2009). DNA methylation in *Acacia mangium* in vitro and ex-vitro buds, in relation to their within-shoot position, age and leaf morphology of the shoot. Silvae Genetica.

[b74] Neale DB, Kremer A (2011). Forest tree genomics: growing resources and applications. Nat. Rev. Genet.

[b75] Nicotra AB, Atkin OK, Bonser SP, Davidson AM, Finnegan EJ, Mathesius U (2010). Plant phenotypic plasticity in a changing climate. Trends Plant Sci.

[b76] Ohri D, Khoshoo TN (1986). Genome size in gymnosperms. Plant Syst. Evol.

[b77] Paun O, Bateman RM, Fay MF, Hedren M, Civeyrel L, Chase MW (2010). Stable epigenetic effects impact adaptation in allopolyploid orchids (*Dactylorhiza: Orchidaceae*. Mol. Biol. Evol.

[b78] Phillips RL, Kaeppler SM, Olhoft P (1994). Genetic instability of plant tissue cultures: breakdown of normal controls. Proc. Natl Acad. Sci. USA.

[b79] Plomion C, Lalanne C, Claverol S, Meddour H, Kohler A, Bogeat-Triboulot MB (2006). Mapping the proteome of poplar and application to the discovery of drought-stress responsive proteins. Proteomics.

[b80] Rabinowicz PD, Citek R, Budiman MA, Nunberg A, Bedell JA, Lakey N (2005). Differential methylation of genes and repeats in land plants. Genome Res.

[b81] Raj S, Bräutigam K, Hamanishi ET, Wilkins O, Schroeder W, Mansfield SD (2011). Clone history shapes *Populus* drought responses. Proc. Natl Acad. Sci. USA.

[b82] Rake AV, Miksche JP, Hall RB, Hansen KM (1980). DNA reassociation kinetics of four Conifers. Can. J. Genet. Cytol.

[b83] Rapp RA, Wendel JF (2005). Epigenetics and plant evolution. New Phytol.

[b84] Rehfeldt GE, Ying CC, Spittlehouse DL, Hamilton DA (1999). Genetic responses to climate in pinus contorta: niche breadth, climate change, and reforestation. Ecol. Monogr.

[b85] Rehfeldt GE, Tchebakova NM, Parfenova YI, Wykoff WR, Kuzmina NA, Milyutin LI (2002). Intraspecific responses to climate in *Pinus sylvestris*. Glob. Change Biol.

[b86] Reinders J, Wulff BBH, Mirouze M, Mari-Ordonez A, Dapp M, Rozhon W (2009). Compromised stability of DNA methylation and transposon immobilization in mosaic *Arabidopsis* epigenomes. Genes Dev.

[b87] Richards EJ (2006). Inherited epigenetic variation – revisiting soft inheritance. Nat. Rev. Genet.

[b88] Richards CL, Verhoeven KJF, Bossdorf O, Wendel JF, Greilhuber J, Dolezel J, Leitch IJ (2012). Evolutionary significance of epigenetic variation. Plant genome diversity Volume I.

[b89] Rival A, Jaligot E, Beule T, Finnegan EJ (2008). Isolation and expression analysis of genes encoding MET, CMT, and DRM methyltransferases in oil palm (*Elaeis guineensis* Jacq.) in relation to the ‘mantled’ somaclonal variation. J. Exp. Bot.

[b90] Rodriguez Lopez CM, Wetten AC, Wilkinson MJ (2010). Progressive erosion of genetic and epigenetic variation in callus-derived cocoa (*Theobroma cacao*) plants. New Phytol.

[b91] Rohde A, Gusta LV, Wisniewski ME, Tanino KK (2009). Bud set – a landmark of the seasonal growth cycle in poplar. Plant Cold Hardiness.

[b92] Rohde A, Junttila O (2008). Remembrances of an embryo: long-term effects on phenology traits in spruce. New Phytol.

[b93] Roudier F, Ahmed I, Bérard C, Sarazin A, Mary-Huard T, Cortijo S (2011). Integrative epigenomic mapping defines four main chromatin states in Arabidopsis. EMBO J.

[b94] Ruttink T, Arend M, Morreel K, Storme V, Rombauts S, Fromm J (2007). A molecular timetable for apical bud formation and dormancy induction in poplar. Plant Cell.

[b95] Santamaria ME, Hasbun R, Valera MJ (2009). Acetylated H4 histone and genomic DNA methylation patterns during bud set and bud burst in *Castanea sativa*. J. Plant Physiol.

[b96] Schellenbaum P, Mohler V, Wenzel G, Walter B (2008). Variation in DNA methylation patterns of grapevine somaclones (*Vitis vinifera* L.). BMC Plant Biol.

[b97] Schmitz RJ, Amasino RM (2007). Vernalization: a model for investigating epigenetics and eukaryotic gene regulation in plants. Biochim. Biophys. Acta.

[b99] Skrøppa T, Johnsen Ø, Mátyás C (2000). Patterns of adaptive genetic variation in forest tree species; the reproductive environment as an evolutionary force in *Picea abies*. Forest Genetics and Sustainability.

[b100] Skrøppa T, Kohmann K, Johnsen Ø, Steffenrem A, Edvardsen ØM (2007). Field performance and early test results of offspring from two Norway spruce seed orchards containing clones transferred to warmer climates. Can. J. For. Res.

[b101] Skrøppa T, Tollefsrud M, Sperisen C, Johnsen Ø (2010). Rapid change in adaptive performance from one generation to the next in *Picea abies*-Central European trees in a Nordic environment. Tree Genet. Genomes.

[b102] Slotkin RK, Martienssen RA (2007). Transposable elements and the epigenetic regulation of the genome. Nat. Rev. Genet.

[b103] Stoehr MU, L'Hirondelle SJ, Binder WD, Webber JE (1998). Parental environment after effects on germination, growth, and adaptive traits in selected spruce families. Can. J. For. Res.

[b104] Thumma BR, Matheson BA, Zhang D, Meeske C, Meder R, Downes GM (2009). Identification of a Cis-acting regulatory polymorphism in a Eucalypt COBRA-like gene affecting cellulose content. Genetics.

[b105] Tsukahara S, Kobayashi A, Kawabe A, Mathieu O, Miura A, Kakutani T (2009). Bursts of retrotransposition reproduced in Arabidopsis. Nature.

[b106] Valledor L, Hasbún R, Meijón M (2007). Involvement of DNA methylation in tree development and micropropagation. Plant Cell Tiss. Org. Cult.

[b107] Valledor L, Meijon M, Hasbun R, Jesus Canal M, Rodriguez R (2010). Variations in DNA methylation, acetylated histone H4, and methylated histone H3 during *Pinus radiata* needle maturation in relation to the loss of in vitro organogenic capability. J. Plant Physiol.

[b108] Van Steensel B (2011). Chromatin: constructing the big picture. EMBO J.

[b109] Verhoeven KJ, Jansen JJ, Biere PJ, van Dijk A (2010). Stress-induced DNA methylation changes and their heritability in asexual dandelions. New Phytol.

[b110] Vining KJ, Pomraning KR, Wilhelm LJ, Priest HD, Pellegrini M, Mockler TC (2012). Dynamic DNA cytosine methylation in the *Populus trichocarpa* genome: tissue-level variation and relationship to gene expression. BMC Genomics.

[b111] Viswanath V, Albrechtsen BR, Strauss SH (2012). Global regulatory burden for field testing of genetically modified tress. Tree Genet. Genomes.

[b112] Webber J, Ott P, Owens J, Binder W (2005). Elevated temperature during reproductive development affects cone traits and progeny performance in *Picea glauca - engelmannii* complex. Tree Physiol.

[b113] Whittle CA, Otto SP, Johnston MO, Krochko JE (2009). Adaptive epigenetic memory of ancestral temperature regime in *Arabidopsis thaliana*. Botany.

[b114] Wilkins O, Waldron L, Nahal H, Provart NJ, Campbell MM (2009). Genotype and time of day shape the *Populus* drought response. Plant J.

[b115] Yakovlev IA, Fossdal CG, Johnsen Ø (2010). MicroRNAs, the epigenetic memory and climatic adaptation in Norway spruce. New Phytol.

[b116] Yakovlev IA, Asante DKA, Fossdal CG, Junttila O, Johnsen Ø (2011). Differential gene expression related to an epigenetic memory affecting climatic adaptation in Norway spruce. Plant Sci.

